# Surgery Advances in Gynecologic Tumors: The Evolution and Outcomes of Robotic Surgery for Gynecologic Cancers in a Tertiary Center

**DOI:** 10.3390/curroncol31050179

**Published:** 2024-04-24

**Authors:** David Knigin, Yoav Brezinov, Shannon Salvador, Susie Lau, Walter H. Gotlieb

**Affiliations:** 1 Division of Gynecologic Oncology, Jewish General Hospital, McGill University, Montreal, QC H3T 1E2, Canada; david.knigin@mcgill.ca (D.K.); shannon.salvador@mcgill.ca (S.S.); susie.lau@mcgill.ca (S.L.); 2 Segal Cancer Center, Sir Mortimer B. Davis Institute of Medical Research, McGill University, Montreal, QC H3T 1E2, Canada

**Keywords:** robotic surgery, cost-effective, gynecologic oncology, innovation

## Abstract

The integration of innovation into routine clinical practice is faced with many challenges. In 2007, we received the mandate to evaluate how the introduction of a robotic program in gynecologic oncology affected patient-centered care by studying its impact on clinical outcomes and hospital resource utilization. Here we summarize the history and experience of developing a robotic surgery program for gynecologic cancers over 16 years. Analysis of the data indicates that robotic surgery improved perioperative patient clinical parameters, decreased blood loss, complications, and hospital stay, maintained the oncologic outcome, and is cost-effective, resulting in it becoming the dominant surgical approach in gynecologic oncology in a tertiary cancer care institution.

## 1. Introduction

In this review on robotic surgery for gynecologic cancers, we will address the evolution faced in the development of a robotic program in gynecologic oncology (gyn-onc) at a tertiary cancer center. The introduction of the robot at the hospital in 2007 created major controversy associated with significant resistance of many members of the departments of surgery and anesthesia. As a result, we received the mandate from the CEO to assess the value and outcomes of performing surgery using the novel robotic platform. Robotics was approved by the FDA for gynecologic surgery in 2005, but there was little experience with the platform to treat gynecologic cancers. There was a strong feeling amongst many of our colleagues, both at the hospital and in our community, that this was an expensive way of performing surgery, not relevant, and not necessary. We took up the challenges and focused on data, both clinical and financial, in order to evaluate the value of robotics. We started by evaluating perioperative outcomes by comparing the cohort of patients immediately before and immediately after the introduction of the robotic platform. Once the data confirmed perioperative improvement, we went on to evaluate the outcomes in the patients at highest risk for surgery, followed by the financial implications, and as soon as survival data became available, we showed that patients who underwent robotic surgery had an overall survival at least as good as the population undergoing a mix of laparoscopy and laparotomy prior to the introduction of robotic surgery.

## 2. Endometrial Cancer

**Figure 1:**
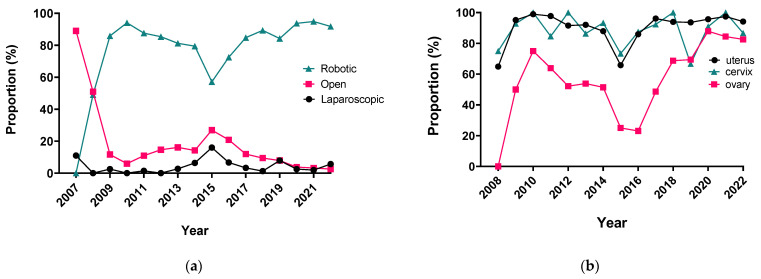
Robotic surgery in gynecologic oncology department in our institution: (**a**) the proportion of three main surgical approaches per year since the introduction of the robotic program; (**b**) the proportion of surgeries that were accomplished robotically stratified by three major cancer sites.

Treatment of endometrial cancer represented a significant proportion of gyn-onc practice when we initiated the robotic program. The integration of robotic surgery in our center was reflected in the rapid switch to minimally invasive surgery (MIS) for endometrial cancer, going from the common rate of 15–20% in most North American institutions in 2007 to offering robotic surgery as a form of MIS to 2/3 of patients within one year and over 90% of patients within two years (Figure 1). This rate has reached close to 100%, with virtually every patient who is eligible for surgery being offered and undergoing robotic surgery. Early adoption of the robotic program resulted in the accumulation of short- and long-term follow-up data evaluating the safety of robot-assisted surgery (RAS). In a pilot study, we reported the outcomes of the first 100 consecutive patients aged 39 to 93 years. During the transition period, we noticed relatively long operative times (247 min) [1], but neither surgical [2] nor anesthesia [3] red flags were identified in the peri- and postoperative complications. Consequently, the gained experience helped develop techniques that improved surgery and oncologic safety. These included positioning, the use of a manipulator placed in the vaginal fornices without an intra-cervical or intra-uterine component, sealing of the fallopian tubes, bagged specimen removal, and the closure of the vaginal vault [4,5]. RAS had inspired the team to search for new and effective training for the fellows and the residents. The DaVinci virtual reality practice modules improved the novices’ robotic technical skills to work from the console, quickly reaching the levels of experienced surgeons [6,7].

### 2.1. Endometrial Cancer: High-Risk Populations

Following our initial findings, we were particularly interested in whether patients at high risk for surgery were equally benefiting from the introduction of robotics. Two populations of patients were studied in further detail, the elderly and patients with elevated BMI.

(A) Patients with elevated BMI

Women with elevated BMI pose both surgical and anesthetic challenges. From the anesthetic aspect, both obesity-related physiological changes and the associated comorbidities increase complications [8]. The number of wound infections, thromboembolic events, excessive blood loss, increased operative times, and prolonged hospital stays is more prevalent in obese patients [9,10]. When operating on obese women, the visibility of the surgical field can be significantly compromised [9]. The use of MIS in obese populations is associated with decreased complication rates and costs [11,12]. However, some of the benefit is lost when conversion to laparotomy is required. BMI is a major risk factor for intraoperative conversion to laparotomy and, as indicated in the randomized LAP-2 trial, may reach above 50% when the BMI is above 40 kg/m^2^ [13]. In our pilot study on the outcome of robotic surgery in obese patients, there were no conversions to laparotomy amongst 33 obese (23 morbidly obese) compared to non-obese (*n* = 52) patients [14]. Yet, short low abdominal incision (mini-laparotomy) for specimen extraction is sometimes necessary (2/23 cases in [14]). The findings were updated this year to include a comparison between 753 obese and 576 non-obese patients. The conversion rate was 0.7–1.0% and similar between the groups. The intra- and postoperative complications were low and similar among the BMI groups [15].

As a comparison, a meta-analysis by Cusimano et al. [16] summarized 46 studies reporting outcomes for laparoscopic and robotic surgeries between the years 2000 and 2018. The pooled conversion rate for robotic and laparoscopic surgeries for patients with BMI ≥ 40.0 kg/m^2^ was 3.8% (eight studies) and 7.0% (nine studies), respectively. In the robotic surgery group, only one study had a conversion rate above 10% [17]. A planned randomized trial that compares the conversion rates for robotic and laparoscopic surgery in obese patients with early endometrial cancer (RObese, NCT05974995) is now ongoing.

Overall, it appears that robotics allows for a reduction in the risk of surgery for obese patients to reach the level of risk observed in non-obese patients. The proportion of morbidly obese women who avoid laparotomy is consistently higher with robotic compared to laparoscopic surgery (Table 1).

(B) Elderly

Elderly patients (≥70 y) require special consideration. Although age is a risk factor for worse outcomes, functional reserve appears to be a better predictor of oncological and surgical outcomes. The term frailty amalgamates multiple physiologic, cognitive, and psychosocial parameters to estimate the individual’s ability to respond to stressors [19,20]. In our center, geriatric assessment is integrated as the standard of care for all patients over 65 years. Compared to the historical cohort (laparotomy and laparoscopy), elderly patients with endometrial cancer who underwent RAS had significantly shorter hospital stay (3 versus 6 days, *p* < 0.0001), decreased blood loss (75 mL versus 334 mL, *p* < 0.0001), but increased surgery duration (244 min versus 217 min, *p* < 0.01). Back in 2014, we published our initial experience with the elderly population. The proportion of perioperative major complications (Clavien–Dindo III-IV) was comparable between elderly and younger patients, while patients who underwent RAS had fewer wound complications, transfusions, and ileus, but 2% required re-operation for bowel injury and a hematoma [21]. To better understand how age impacts the outcome of RAS for endometrial cancer, 197 non-elderly (ages 38–69 years) patients were compared to 106 elderly patients (ages 70–93 years) with endometrial cancer. Elderly patients had higher stage, grade, and more comorbidities. Patients over the age of 80 had more Clavien–Dindo grade III and IV complications (12/106 vs. 2/197, *p* < 0.0001) [22]. More than 90% of patients up to the age of 80 y left the hospital within 2 days, and up to 50% of patients above 80 y left within 3 days. Further re-evaluation in 2023 on 1216 robotic surgeries for endometrial cancer found that 1/3 of the patients were 70 years or older, and these had a 5-year cancer-specific survival of 93.2% versus 96.6% in the younger patients (Racovitan F., in preparation).

As a comparison, a meta-analysis of five retrospective studies compared the outcomes of robotics versus laparotomy for patients over 65 years of age [23], with one study contributing 93.6% of the study population [24]. There were 1472 robotic surgeries and 6130 laparotomies. The relative risk (RR) for major complications was 0.42 (*p* = 0.002), and the pooled difference in the length of stay was −3.34 (95% CI −4.36 to −2.31) in favor of robotic surgery [23].

### 2.2. Endometrial Cancer: Sentinel Lymph Nodes

Based on the data from Abu Rustum et al. [25], we started to perform sentinel node assessment for endometrial cancer in 2010. Transitioning to the sentinel lymph node protocol required repeated internal quality assessments. We shifted over from using three tracers, methylene blue, technetium-99 (99mTc), and indocyanine green (ICG) [26], to using two tracers following a prospective randomized study showing the absence of the value of methylene blue [27], and we presently use only ICG. The added value of SLN in endometrial cancer is hypothesized to result from the increased detection of metastatic disease, either from positive SLN detected outside the traditional areas covered by lymphadenectomy (incidence 7.5–20%) or the ultrastaging detecting micrometastases that otherwise would have been missed by conventional pathology staining (18.5–25.7%) [28,29]. As anticipated, the better identification of the involved lymph nodes along the pelvic sidewalls and subsequent optimized planning of adjuvant treatment led to decreased pelvic sidewall recurrences. We compared 250 patients with SLN assessments to 193 patients who underwent standard lymphadenectomy for endometrial cancer, and with a median follow-up of 6.9 years, we found increased overall and progression-free survival when SLN was performed [30].

In many countries, patients with a diagnosis of grade 1 endometrial cancer are not operated on by gynecologic oncologists and routine lymph node assessment is not performed. In addition, the role of routine lymph node assessment in endometrial intraepithelial neoplasia (EIN) is controversial. As the robotic approach had significantly facilitated the use of sentinel node detection by the ICG tracer, we began to perform a prospective study evaluating the value of routine lymph node evaluations in both grade 1 endometrial cancer and EIN in 2014. The analysis of the clinical benefit of such an approach showed a 12.3% change in the assignment of adjuvant treatment in preoperative grade 1 endometrial cancer (Knigin D., in preparation) and 6.8% in EIN [31]. Until innovative molecular analyses will allow for the better triage of patients benefiting from adjuvant therapy, sentinel node sampling appears to benefit these patients.

## 3. Cervical Cancer

Cervical cancer is less common in developed countries, and the experience of a single center is often limited [32]. In a pilot study, we evaluated 16 radical robotic hysterectomies performed between 2008 and 2009 and compared them to the immediate past 24 radical hysterectomies performed by laparotomy between 2003 and 2007, just prior to the introduction of robotics in our institution. We went over from 100% laparotomy to 100% robotic surgery for cervix cancer, and this was associated with decreased blood loss (106 vs. 546 mL), faster resumption of diet (1.2 vs. 3.5 days), decreased length of stay (1.9 vs. 7.2 days), and less wound complications (0% vs. 29%). The robotic approach took 68 min longer during the learning curve [32]. Following the presentation of the LACC trial in 2018 [33], we reviewed the outcome of the 74 robotic radical hysterectomies we had performed. The operative time was 282 min, comparable to the duration of open radical hysterectomies [34]. The rest of the outcomes remained similar to the early report, suggesting a fast and stable improvement in the perioperative outcomes. The rate of recurrence after robotic radical hysterectomy was comparable to the laparotomy cohort in our institution. This suggested that patient selection might be the key to the successful implementation of MIS in cervical cancer. In addition, our results can be explained by some precautionary measures. We never placed any intra-cervical or intra-uterine devices, we oversewed the exposed tumor, and performed specimen removal in bags placed via the vagina at the end of surgery to avoid difficult extractions and manipulation of the specimen, thus avoiding the potential spillage of cancer cells.

In this context, a recent randomized controlled trial (SHAPE) showed non-inferiority of simple vs. modified radical hysterectomy in low-risk cervical cancer (<2 cm, depth < 10 mm). In the radical hysterectomy arm, pelvic recurrence was similar between MIS and open surgery arms [35]. Yet, a review of the published literature concluded that until further evidence is available, the gold standard surgical approach for early cervical cancer should remain open radical hysterectomy [36]. Two randomized controlled trials are presently evaluating the safety of robotic radical hysterectomy in early cervical cancer. The US-led ROCC/GOG-3043 (NCT04831580) is testing the hypothesis of the non-inferiority of the robotic approached compared to the open approach. The primary end-point is 3-year disease-free survival. The RACC trial (NCT03719547), led by the Karolinska institute in Sweden, is of a similar design and will follow-up the patients for 60 months.

## 4. Ovarian Cancer

Coinciding with the integration of the robotic platform in our center, we observed that many patients who underwent interval cytoreduction had limited disease that could be amenable for complete resection using RAS. We performed a prospective case study of robotic interval cytoreduction surgery in carefully selected patients. The decision to proceed with robotic cytoreduction was made by the department’s multidisciplinary tumor board. Between 2008 and 2014, 62.6% of interval cytoreductions were performed by robotics. This approach was compared to a historic cohort of 22 laparotomy cases [37]. We realized that there was a selection bias as the historic cohort included higher-risk patients, as reflected in the lower complete cytoreduction rate (40.9%) compared to the study period (75.8%). Also, the proportion of primary cytoreductive surgeries (PCSs) for advanced ovarian cancer declined during the study period. With this in mind, we saw a 46% decrease in surgical blood loss and a trend toward decreased transfusion (60% vs. 38.5%, *p* = 0.087) in the “robotic era” compared to the “pre-robotic era”. The progression-free survival and overall survival were similar between the groups. When the robotic surgery patients were plotted separately, their progression-free interval and survival were significantly higher than those of laparotomy-treated patients. We have currently expanded our prospective cohort to over 250 patients, and a conservative assessment at this time is that with careful selection, a significant proportion of patients with ovarian cancer can benefit from minimally invasive procedures.

As a comparison, the feasibility of robotic-assisted cytoreductive surgery after neoadjuvant therapy has further been demonstrated in retrospective studies [38,39] and is currently being investigated in a phase III non-inferiority randomized controlled trial—LANCE [40].

We have started to investigate the SLN approach in ovarian cancer. Technically, the dye is injected into the utero-ovarian or infundibulopelvic ligaments. In a short surgical video, one can observe how the ICG is directed by the surgeon using a “spinal” needle introduced through the anterior abdominal wall [41]. At present, one out of three prospective studies that investigate the accuracy of SLN in early ovarian cancer completed accrual and are pending publication (SELLY study, NCT03563781).

## 5. Patient-Reported Outcomes

Since the initiation of the robotic program in our center, we studied the impact of robotic surgery on the health-related quality of life (HRQoL) of endometrial cancer patients. A descriptive pilot study of the first 109 consecutive patients found that 61.5% of patients had no postoperative pain one week after surgery, and the time to the resumption of typical daily activities was 11 days [42]. We identified a significant decline in anti-inflammatory and narcotic analgesics use, and 75% did not use any postoperative narcotics. In the follow-up study including 211 gynecological cancer patients, we prospectively assessed the HRQoL using a validated FACT g questionnaire [43]. The HRQoL score on the physical and functional domains decreased by less than 10% one week after surgery and had returned to baseline at 3 weeks. Emotional well-being was found to increase after surgery and remain significantly higher than baseline.

## 6. Hospital Resource Utilization

The assessment of the implementation of a new technology in healthcare extends beyond safety and clinical effectiveness. Both the costs of medical care and the impact on the departmental patient flow require evaluation. A new technological solution would optimally improve patient outcomes or lower the cost of care, preferentially achieving both. In our institution, the implementation of robotic-assisted surgery has increased the proportion of MIS from 11% to 90% (2007 to 2010) of all surgeries (excluding vulvar cancer, Figure 1).

Presently, virtually all uterine and cervix cancer cases, and most interval cytoreductions in our department are performed robotically. We calculated the hospital costs for each major gynecological cancer intervention [2,32,44,45]. We reported a 26% decrease in hospital costs per event of care (procedure + admission) for endometrial cancer staging following the initiation of the robotic program [2]. The increased equipment costs are mitigated mainly by a shorter length of stay (≤1 days compared to 5 days) and the low conversion rate to laparotomy, which is <1% for endometrial cancer (*n* = 1329, 22% were BMI > 40 kg/m^2^)^30^.

Radical hysterectomy for cervical cancer using robotics is financially justified, with a cost saving per case of about CAD 2000 to 3000 depending on amortization schemes [32]. The proportion of patients undergoing MIS surgery for ovarian cancer increased from 0% to 58% following the introduction of the robotic program [44]. The average cost of RAS for ovarian cancer was 43% lower than the same case performed by laparotomy. This was attributed to a shorter length of stay and less complications following RAS.

As a comparison, Table 2 provides a synthesis of pertinent studies and their respective outcomes. Potential avenues for cost reduction in robotic surgeries include diminished procurement expenses through the purchase of multiple platforms per institution, increased utilization of the robotic platform measured in cases per year [46], augmented surgeon proficiency resulting in reduced surgery durations and fewer conversions to laparotomy [47], and competition among manufacturers that would lead to competitive prices. Beyond cost-effectiveness, the introduction of robotics had a profound effect on resource allocation on the surgical ward. We found that 5 years post-first robotic surgery, the annual number of elective surgeries increased by 27%, while the total surgery-related admission days dropped by 42% (36% to 21%) [48]. Altogether, the introduction of robotics allowed for resource allocation toward more complex patients without increasing costs.

## 7. Future Perspective

The robotic system introduces a computer interface between the surgeon and the patient. At present, this already allows for virtual training, improved three-dimensional immersion images, the elimination of tremor, and feedback to the surgeon about their performance on specific parts of the surgery compared to their previous selves and to their peers [57]. The cyborg-equivalent robotic platform has the potential to enable the amalgamation of surgical skills with digital analysis, machine learning, and artificial intelligence. The foreseeable improvements in the robotic platform are substantial and include various prototypes presently under investigation. At the frontline, there is the integration of radiologic imaging, such as computed tomography or magnetic resonance imaging, into the surgical field. It aims to facilitate the accurate localization of surgical targets and can be invaluable when the targets are small or the anatomy is obscured (e.g., retroperitoneal fat, adhesions) [58]. Real-time image processing and multispectral analysis of the surgical field assist with tissue classification, allowing us to identify cancers and create “no fly zones” to prevent injury to healthy tissues [59]. While current systems lack haptic feedback, the novel fifth-generation robotic platform from Intuitive, approved by the FDA in March 2024, does provide haptic feedback.

## 8. Conclusions

The integration of a novel technology into routine clinical practice is associated with many challenges. Supported by research and validation, robotic surgery became safer, cost-effective, and the dominant approach in gynecologic oncological surgery in several institutions. Innovation in medical care is a responsibility, and akin to the introduction of new drugs and experimental treatments, the introduction of enhanced state-of-the-art surgical tools ensures the delivery of the best possible patient-centered care.

## Figures and Tables

**Table 1:** Perioperative outcomes in patients with BMI ≥ 40.0 kg/m^2^.

Cohort	Conversion	Intraop. Complications ^1^	Hospital Stay
Robotic			
The author’s cohort	1.0%	6.6%	90.3% **< 2 d**
Cusimano et al. [16]	3.8%	NA	NA
Lechartier et al. [18]	4.9%	2.2%	1.1 d (mean)
Laparoscopy			
LAP-2 trial [10,13]	57.1%	8.2–9.8%	48% **< 2 d**
Cusimano et al. (2019) [16]	7.0%	NA	NA

^1^ Abdominal vessel, nerve, or viscus injury. Abbreviation: NA, data not available.

**Table 2:** Studies estimating costs of robotic surgery.

Study	Region and Years of Application	Single vs. Multicenter	Cancer Type	Outcome for Overall Costs
Leitao M. [49] (2014)	USA 2007–2010	S	Endometrial	RS > LS
Marino P. [50] (2015)	France 2007–2010	M	Cervical Endometrial	RS > LS, increased saving with more procedures/year
Chan JK. [51] (2015)	USA 2011	M	Endometrial	RS > OS > LS BM ≥ 40 kg/m^2^
Bogani G. [52] (2016)	USA 2007–2012	S	Endometrial	Same as open 30 d costs
Korsholm M. [53] (2019)	Denmark 2007–2012	M	Endometrial	RS = OS/LS for obese and RS > OS/LS for non-obese
Casarin J. [54] (2020)	USA 2013–2015	M	Endometrial	RS = OS, costs up to 30 days post-op
Kosa SD. [55] (2021)	Canada 2012–2014	M	Endometrial	BMI ≥ 40 kg/m^2^ RS = OS = LS
Yoon JH. [56] (2024)	Republic of Korea 2010–2020	S	Endometrial	RS > LS

Abbreviations: LS, laparoscopic surgery; OS, open surgery; RS, robotic surgery.

## Data Availability

Data supporting reported results can be found in the peer reviewed articles referred to in the manuscript.
